# Progress in Diseases of the Nervous System

**Published:** 1904-04-23

**Authors:** 


### Progress in Diseases of the Nervous System.

**Tuberculosis of the Nervous System.**—The Bradshaw Lecture by Trevelyan [1] dealt with the various forms of tuberculosis of the nervous system. He thinks it doubtful whether it is possible to have a tuberculous meningitis without the presence of tubercles, though at post-mortem examinations few, if any, are to be found in some cases. A limited meningitis may occur, consisting of a thickening of the membranes, with tubercle formation, over a limited portion, usually of the motor cortex; its symptoms differ from those of ordinary tuberculous meningitis. Tuberculous masses are frequent, either single or multiple, varying in size from a millet-seed up to an orange. They are surrounded by an area of vascular engorgement, but as a rule there is no definite capsule. The centre is caseous, with a peripheral growing zone of recent tuberculous tissue. Except in the medulla, pons, and cord, where they are usually surrounded by nervous tissue, they most often project on the surface. The infection of the cerebro-spinal fluid is thus easily brought about, and in two-thirds of the cases a tuberculous meningitis is also present. A complete post-mortem examination almost always reveals an older tuberculous focus somewhere in the body. Infection may spread from a neighbouring focus—*e.g.* the nose, ear, bone disease—though not very commonly. The lungs may be the seat of miliary tubercle or of phthisis; the abdomen may show tuberculous peritonitis or intestinal ulceration. Lymphatic glands are very often found affected, particularly the mediastinal ones. Thus in 40 cases (35 with miliary tuberculosis) no old focus was found other than the adenopathy. This close relationship is connected with the fact that the lungs constitute the port of entry of the tubercle bacilli in the large majority of cases. The route by which the bacilli travel from the infecting focus is probably the blood vessels and not the lymphatics, for the former are often found affected, and experimentally it is possible to produce a meningitis by injection of bacilli into the carotid artery, but not by injection into the lymphatics. Examination of the spinal fluid, obtained by lumbar puncture, is an important addition to our means of diagnosis. Bacilli and lymphocytes may be found by microscopical examination of the deposit, or the fluid may be used for an inoculation experiment. Recovery from tuberculous meningitis exceptionally occurs, but the prognosis, for all practical purposes, is hopeless. In cases of tuberculous tumour, recovery, or rather subsidence of symptoms, is perhaps a little more common.

**Syringal Hæmorrhage into the Spinal Cord.**—Gowers [2] relates four cases in which hæmorrhage occurred into pre-existing cavities in the spinal cord. The condition of syringomyelia is a congenital defect of the spinal cord characterised either by a cavity in the middle line, usually continuous with the central canal, or by a cavity adjacent to the posterior horn. These cavities are usually bordered by undeveloped embryonal tissue, which may in some cases grow, forming a gliomatosis of the spinal cord. Again, glioma of the pons is sometimes connected by tracts of similar tissue with gliosis of the cervical cord. Developmental defects may thus extend up from the cord to the mid-brain, though seldom as actual cavities. Distension of such cavities may slowly come on after adult life is reached, producing characteristic symptoms—pain, analgesia without anæsthesia, and muscular wasting when the anterior grey substance is reached. Sudden hæmorrhage may occur into these cavities and produce curious symptoms. Thus in one case, a man, aged 40, suddenly developed paralysis of the left arm, preceded by sudden pain in the right side of the head and giddiness. The paralysis was both of movement and sensation. Renal and cardiac disease were absent, and yet the cause was hæmorrhage, as shown by the sudden onset. The hæmorrhage was not cerebral, for a cerebral lesion never produces complete paralysis of the arm, without any affection of the face or leg, owing to the contiguity of the cortical areas and pyramidal tracts. Further, there was complete loss of power of the trapezius, sterno-mastoid, and of the pectoralis of the left side—a condition which is not found in cerebral paralysis. Also the loss of sensibility was limited to the arm, shoulder, and side of the neck, whereas on the face, head, and abdomen sensibility was normal. Further examination showed a congenital defect of movement of the eyes, of both eyes to the right, and of almost all movements of the left eye. This pointed to a congenital defect in the upper part of the pons on the left side. This revealed the probability of a state of the cord such as might have determined both the occurrence and the limitation of the disease—a congenital cavity into which hæmorrhage had occurred. The paralysis slowly improved, and recovery ensued in the course of two years. A corroboration of the diagnosis was afterwards afforded by the subsequent occurrence of Charcot's arthropathy in the left hip joint, a condition which is found in syringomyelia as well as in tabes.

**Tabes in the Negro.**—Four cases are recorded by Hecht,[3] who states that, in his opinion, Aryan admixture is essential to its production. In each case inquiry elicited that the patient's ancestry was not of pure negro blood. Hecht further says that a black negro need not necessarily be a pure-blooded negro. The rarity of tabes, in view of the large amount of syphilis amongst negroes, is a striking fact and as yet an unexplained one. Tabetics with optic atrophy are rarely ataxic, and conversely ataxics rarely become blind. The explanation of this, according to Edinger and Marie, lies in the fact that blind men rather conserve than dissipate muscular energy. They do not over-exercise. With them functional wear and tear is at a minimum, and so the disease in the cord is restricted.

**Facial Paralysis.**—Jacoby[4] records some cases of peripheral facial paralysis bearing on the question of the involvement of the orbicularis palpebrarum. It is usually stated that, whereas in central (supranuclear) facial paralysis the orbicularis is not affected, all branches of the nerve are involved in the peripheral type. More recent observations have shown that neither of these statements is quite true. In regard to the former class of case, it has been found that, though the patient is able to close both eyes together, he is not able to shut the eye on the paralysed side alone. The explanation of this lies in the fact that movements which habitually take place bilaterally are innervated from both sides of the cortex, whereas those which are unilateral are innervated only from the opposite cortex. And it has been experimentally shown that fibres decussate across the median raphé between the facial nuclei. In peripheral facial paralysis Jacoby finds that, though at first the orbicularis palpebrarum is paralysed, yet during recovery a stage occurs in which the muscle recovers before the other facial muscles, so that the patient, though not able to shut the affected eye alone, is able to do so when told to shut both eyes.

**Herpes Zoster and Facial Paralysis.**—Frazer[5] reports the case of a man who had an attack of right-sided herpes zoster, followed five days later by facial paralysis. The herpes affected the skin innervated by the third division of the fifth nerve and by the second, third, and fourth cervical nerves on the right side. The facial palsy was of peripheral type and taste was dulled; hearing was good both for low- and high pitched notes. This last-mentioned fact shows that it was not the geniculate ganglion which was affected, but the nerve below that point.

**Acromegaly and Gigantism.**—The relationship of these two diseases is the subject of an interesting discussion by Bassoe.[6] All authors agree that giants frequently present acromegalic symptoms, and that hypophysis disease is the rule in acromegaly. Woods Hutchinson states that in gigantism the greater part of the overgrowth is found at or near the tips of the segment crescents, as in acromegaly, differing from the latter mainly in that it is not exclusively confined to the tip of the segment or last division of the limb. The facial part of the skull is enlarged out of all proportion to the cranial, particularly in the lower jaw. The condition is one which tends to shortness of life, and the individual has little mental, physical, or sexual vigour. Acromegaly and gigantism are simply different expressions of one and the same condition: acromegaly is a general overgrowth tendency which does not begin to express itself until after adult structure is reached, and which consequently expends itself on those points in the body at which growth last ceased—the extremities of the segment crescents and the distal extremities of the appendages; gigantism in the large majority of cases is this same condition manifesting itself in childhood or before complete stature has been reached, and the growth in consequence is more symmetrical and less strictly confined to the last segment of the arches and the appendages. Disturbances of the function of the pituitary body are the principal factors in both conditions. The nature of the overgrowth in both these diseases is primarily a pure functional hypertrophy; later, immature tissue of a mixed type is formed or cysts are produced. Sternberg states that 20 per cent. of all acromegalics are tall, and that about 40 per cent. of all giants have acromegaly, but denies that the two processes are identical. He believes that there are normal giants—*i.e.* well-proportioned overgrown people. He classes pathological giants as follows: (1) Acromegalics, about 40 per cent.; (2) cases with multiple tumour-like exostoses (leontiasis ossea and hyperostoses); (3) cases with facial hemihypertrophy; (4) cases with multiple curvature of the bones. Sternberg sees two possibilities for an etiological relationship between gigantism and acromegaly. The latter may induce a cartilaginous proliferation in a still ununited epiphysis, or in gigantism a disposition to general dystrophies, and particularly to acromegaly, may exist.

[1] Brit. Med. Jour., Nov. 7. 1903. [2] Lancet, Oct. 10, 1903. [3] Amer. Jour. Med. Sci., Oct. 1903. [4] Jour. Nerv. and Ment. Dis., Oct. 1903. [5] Lancet, Jan. 2, 1904. [6] Jour. Nerv. and Ment. Dis., 1903.

